# Biomolecular Perturbations in In-Cell Dynamic Nuclear Polarization Experiments

**DOI:** 10.3389/fmolb.2021.743829

**Published:** 2021-10-21

**Authors:** Sarah A. Overall, Alexander B. Barnes

**Affiliations:** Laboratory of Physical Chemistry, ETH Zürich, Zürich, Switzerland

**Keywords:** in-cell NMR, dynamic nuclear polarization, in-cell DNP-NMR, cryopreservation, apoptosis, cell viability

## Abstract

In-cell DNP is a growing application of NMR to the study of biomolecular structure and function within intact cells. An important unresolved question for in-cell DNP spectroscopy is the integrity of cellular samples under the cryogenic conditions of DNP. Despite the rich literature around cryopreservation of cells in the fields of stem cell/embryonic cell therapeutics, cell line preservation and in cryo-EM applications, the effect of cryopreservation procedures on DNP parameters is unclear. In this report we investigate cell survival and apoptosis in the presence of cryopreserving agents and DNP radicals. We also assess the effects of these reagents on cellular enhancements. We show that the DNP radical AMUPol has no effect on membrane permeability and does not induce apoptosis. Furthermore, the standard aqueous glass forming reagent, comprised of 60/30/10 d_8_-glycerol/D_2_O/H_2_O (DNP juice), rapidly dehydrates cells and induces apoptosis prior to freezing, reducing structural integrity of the sample prior to DNP analysis. Preservation with d_6_-DMSO at 10% v/v provided similar DNP enhancements per √unit time compared to glycerol preservation with superior maintenance of cell size and membrane integrity prior to freezing. DMSO preservation also greatly enhanced post-thaw survival of cells slow-frozen at 1°C/min. We therefore demonstrate that in-cell DNP-NMR studies should be done with d_6_-DMSO as cryoprotectant and raise important considerations for the progression of in-cell DNP-NMR towards the goal of high quality structural studies.

## Introduction

In-cell NMR is the only technique that can provide structural, dynamic, and composition information of biomolecules in their native, intracellular context and at atomic resolution. However, cellular heterogeneity leads to spectral complexity and reduced sensitivity to spins of interest. These challenges are at the forefront of driving NMR method development. To date, in-cell NMR has yielded *de novo* in-cell structures of GB1 in eukaryotic cells (Muntener et al., 2016; Pan et al., 2016) and TTHA1718 in *E. coli* (Sakakibara et al., 2009), established interactions between *α*-synuclein and cellular chaperones (Burmann et al., 2020) and monitored structural maturation of SOD1 during protein folding *in situ* (Banci et al., 2013). Much of this work was pioneered by the work of Selenko and co-workers who demonstrated the transfer of high concentrations of uniformly labelled proteins into cells using electroporation and microinjection (Selenko et al., 2006). This circumvents some of the spectral complexity observed in in-cell NMR spectra. However, this requires very high concentrations of exogenously produced soluble proteins, which is not applicable to membrane proteins, transient protein complexes, proteins which can’t be concentrated or recombinantly produced. In addition, highly elevated cellular concentrations of the protein of interest are established, which for most proteins is non-physiological. The detection of physiologically relevant protein systems requires significant improvements in NMR sensitivity. Dynamic nuclear polarization (DNP) is one such technology. In-cell Dynamic Nuclear Polarization Nuclear Magnetic Resonance (DNP-NMR) holds great promise for physiologically relevant biomolecular structure determination, particularly for membrane bound proteins but also for the study of a wide range of cellular phenomena at the atomic level as demonstrated by in-cell NMR studies in both solution and solid state (Theillet et al., 2016; Thongsomboon et al., 2018; Overall et al., 2019; Luchinat et al., 2020). The sensitivity gains from DNP give access to many dilute biomolecular systems that are otherwise inaccessible to structural and compositional studies within the physiological context of an intact cell. The first demonstration of in-cell DNP within intact mammalian cells showed that significant enhancements could be achieved in the context of intact cells with a range of polarizing agents (Albert et al., 2019), with an added benefit that the DNP enhancement can be targeted to subcellular compartments. Subsequent studies used DNP-NMR to observe exogenously labelled ubiquitin electroporated into HeLa cells (Narasimhan et al., 2019), drug mediated expression of HIV particles (Overall et al., 2020) and antisense RNA drug complexes in HEK 293T cells (Schlagnitweit et al., 2019). However, this potential remains largely untapped. Optimization of sample preparation and an understanding of the cellular effects of DNP preparation are lacking.

The study of cryopreservation is a rich and active field of research that has extensively explored many different cryopreserving reagents and formulations since the birth of cryobiology in the 1950s. Cooling methods have also been explored to improve the storage of cells and tissue for research and therapeutic purposes (Raju et al., 2021) (Hornberger et al., 2019). Much of the reported work is centered around reducing intracellular ice crystal growth, considered to be the most damaging effect of cryogenic preservation (Mazur 2004). Vitrification, the formation of a non-crystalline amorphous glass, is an effective method for limiting ice crystal formation and maintaining high cell viability (Fahy and Wowk 2020). Vitrification is generally achieved through rapid cooling and provides the highest resolution of cellular structures by cryo-electron microscopy as well as being the method of choice for blastocyst and embryo preservation in IVF clinics (Sekhon et al., 2018) (Valjerdi et al., 2009). Thus, vitrification is an important method for the cryopreservation of mammalian cells. In reality, cooling rates required to vitrify cellular samples can be difficult or impossible to achieve for samples larger than a few nano liters (Berejnov et al., 2006). As a result, slow-cooling at 1°C/min is commonly used for the long-term storage of cell lines and primary cells for its ease of use (Baust et al., 2009).

Significant dehydration is observed among slow-cooled cryopreserved cells caused by the extraction of water from the intracellular compartment (Mazur 1984). Some have suggested that this dehydration event enhances cell survival by removing water that might otherwise nucleate ice crystals intracellularly (Meneghel et al., 2019). However, cryoprotecting media that maintains cell size by reducing dehydration proves much more effective at preserving cell viability (Huebinger et al., 2016). Molecular adaptations during slow-freezing or through interactions with cryoprotectants are thought to contribute to the ability of cells to withstand and recover from ice crystal growth (Mazur 2004; Meneghel et al., 2019). Dimethyl sulfoxide (DMSO) is an important and widely used cryopreserving agent for mammalian cells, which readily diffuses through cell membranes and appears to particularly enhance the adaptability of cell membranes to ice damage (Shi et al., 2001; Huebinger 2018) in addition to reducing lethal intracellular ice formation (Baust et al., 2009).

Despite this research, in-cell DNP studies have often utilized glycerol based preservation (60:30:10 d_8_-glycerol:D_2_O:H_2_O) and liquid nitrogen flash-freezing for sample preparation. Flash-freezing, typically not adequate to vitrify large sample volumes, has been implemented for in-cell studies due to the reported instability of DNP radicals to the reducing environment of the intracellular compartment by us and others (Giotta and Wang 1972; Albert et al., 2019; McCoy et al., 2019). The use of DNP juice was established by Hall and co-workers, who showed that DNP of biomolecular samples could be greatly improved using high concentrations of glycerol to promote the formation of a homogenous glass (Hall et al., 1997). Ice crystal formation is associated with lower DNP enhancements due to increased paramagnetic quenching as the local concentration of radicals outside of ice crystals increases (Corzilius et al., 2014) further supporting the importance of glass forming reagents. Further studies revealed that heterogenous imperfections of the glassy matrix reduce polarization transfer, even at glycerol concentrations of <55% (Leavesley et al., 2018b). As a result, net DNP enhancements are maximized in homogenous glasses promoted by glycerol in aqueous samples.

Cellular samples are very different. High concentrations of any one molecule are often greatly detrimental to cellular homeostasis, even over short periods of time, creating osmotic imbalances that impose significant stress onto cells as well as other toxic effects (Baust et al., 2009). Furthermore, the distribution of cryopreserving reagents will inherently be heterogenous due to the heterogenous distribution of cellular structures with differing solvent accessibilities. This raises questions about the uniformity of in-cell enhancements and the appropriateness of glycerol based protecting reagents for in-cell studies. Here we perform a study of cellular integrity before and after DNP analysis and assess the effects of different cryoprotecting agents on DNP parameters and spectral quality of Jurkat T cells at natural abundance.

## Materials and Methods

### Cell Culture

Jurkat T cells and a variant of the Jurkat T cell line JLat9.2 cells (containing a genomically integrated HIV genome that is basally inactive (Jordan et al., 2001)) were cultured at 37°C with 5% CO_2_ atmosphere in unlabeled complete RPMI (2 mM L-Glutamine, 10% v/v Fetal Bovine Serum (FBS) (Gibco), 100 U/ml penicillin-100 μg/ml streptomycin (Gibco) and 10 mM sodium pyruvate (Gibco). Cells were counted using a hemocytometer and trypan blue (sigma-aldrich) staining by preparing a ½ dilution of 0.4% trypan blue with cells and observing under a light microscope. Blue cells were counted as dead and non-blue refractive cells were counted as viable.

### Flow Cytometry

1 × 10^6^ total cells were transferred to 3 ml flow cytometry tubes and washed with 1 ml FACS buffer (phosphate buffered saline (PBS), 1% Bovine serum albumin (BSA), 2 mM EDTA). Cells were then incubated with 1:100 diluted BioTracker NucView Caspase-3-405 (Biotium) for 15 min on ice then washed with 1 ml FACS buffer and resuspended in 500 μl FACS buffer prior to analysis. 1 min before analysis 10 μl of 10 μM propidium iodide (PI) was added and the sample analyzed on an LSR Fortessa flow cytometer (BD Biosciences). Data was analyzed with FlowJo software (treestar). All events were analyzed and reported. The only gating used was to remove doublets as shown in Supplementary Figure S1.

### Annexin-V Staining

Annexin-V staining was done after caspase-3 staining. The cells were washed with Annexin-V binding buffer (0.01 M HEPES pH 7.4, 0.14 mM NaCl, 2.5 mM CaCl_2_). Then resuspended in 100 μl Annexin-V binding buffer and 5 μl Annexin-V-488 (Sigma) added to each sample and incubated on ice for 10 min then diluted with 1 ml of Annexin-V binding buffer before immediately analyzing without washing.

### Pre-Freeze Analysis

10 × 10^6^ total cells were transferred to FACS tubes and washed with 2 ml FACS buffer by centrifugation. Cell pellets were then resuspended in 10 μl of cryopreservative as described in the sample preparation section but in the absence of radical. The cells were then incubated on ice for 10 min followed by washing with 1 ml of FACS buffer, resuspended in 100 μl of FACS buffer and stained for caspase-3 and Annexin-V as described above.

### Post-Thaw Cell Culture

Frozen sapphire rotors containing cells were thawed in a 37°C water bath for no more than 5 s. Zirconia caps were immediately removed and the cells were collected by centrifugation upside down in a 15 ml conical tube for 1 min at 1,500 rpm at 4°C. The cells were immediately and gently resuspended in 3 ml prewarmed complete RPMI then transferred to six well plates and cultured for 24 h at 37°C prior to FACS analysis and trypan blue counting.

### DNP-NMR Sample Preparation

Cell samples were prepared by washing 40 × 10^6^ total JLat 9.2 or Jurkat T cells with 5 ml ice cold phosphate buffered saline (PBS) and gently pelleted at 1,500 rpm for 5 min at 4°C to remove culture media. The cell pellet (approximately 40 μl) was resuspended in 1 ml of deuterated PBS and incubated on ice for 10 min to allow exchange of intracellular water with D_2_O. The cells were then pelleted again and resuspended in an equal volume of 2 fold concentrated cryopreserving reagent with DNP radicals. (20% DMSO with 20 mM AMUPol, 60/30/10 glycerol/D_2_O/H_2_O with 20 mM AMUPol or PBS with 20 mM AMUPol), to give a final AMUPol concentration of 10 mM (Sauvee et al., 2013). The cells were then incubated for the indicated times before packing into 3.2 mm sapphire cylindrical rotors (Bruker Biospin) using custom made Teflon filling tools (Overall et al., 2020) by centrifugation for 1 min at 1,500 rpm. Rotors were plunge frozen in liquid nitrogen for 10 min before capping with zirconia drive caps. Rotors were stored in liquid nitrogen prior to DNP analysis.

Slow-frozen cells were prepared as above but after filling, the rotors were immediately capped with zirconia drive caps and slow-cooled in a Mr. Frosty (Nalgene) at −80°C overnight before plunge freezing in liquid nitrogen for storage.

### Solid-State DNP-NMR

Solid-state DNP-NMR was conducted on a 14 T Bruker DNP spectrometer operating at 600 MHz ^1^H Larmor frequency and equipped with a second harmonic 365 GHz gyrotron and a 3.2 mm HX or HXY LTMAS probe. Samples were spun at 9 kHz with a sample temperature of ∼104–108 K (microwaves off) and ∼110–114 K (microwaves on). ^13^C spectra were acquired using a cross polarization (CP) scheme with typical ^1^H spin-locking amplitude of 100 kHz over a 1 ms tangential ramp centered at 50 kHz. Data was acquired under spinal64 ^1^H decoupling at 100 kHz with 512 transients and recycle delay 1.26*T_1_. Enhancements were calculated as both:

The peak intensity ratio: ε_on/off_ = $\frac{Ion}{Ioff}$ and enhancement per √unit time: $\left(\frac{\varepsilon }{\sqrt{TB}}\right)$ . Effective enhancement per √unit time was calculated by: $\left(\frac{\varepsilon }{\sqrt{TB}}\right)\times \;\xi$

The quenching factor ξ was calculated as: $\xi =1-\frac{I}{I_{0}}$ . Polarization build up times (T_B_) were measured using a saturation recovery CP-sequence with 20 × 5 μs saturating pulses prior to the recovery delay.

### Data Analysis

1D NMR data was processed in Topspin 4.07 using 50 Hz line broadening. Cellular components were assigned using data from the BMRB. T_B_ was calculated from peak intensities and fit to the equation:

$$Mz\left(t\right)=Mz\left(1-e^{-\frac{t}{TB}}\right)$$

Only fits with an *R* ^2^ value of >0.97 are reported and used for further analysis. Statistical data was plotted and processed using GraphPad Prism 9. Statistical significance was determined using a parametric unpaired t test.

## Results

### Pre-Freeze Viability of Cells Prepared for DNP

**FIGURE 1:**
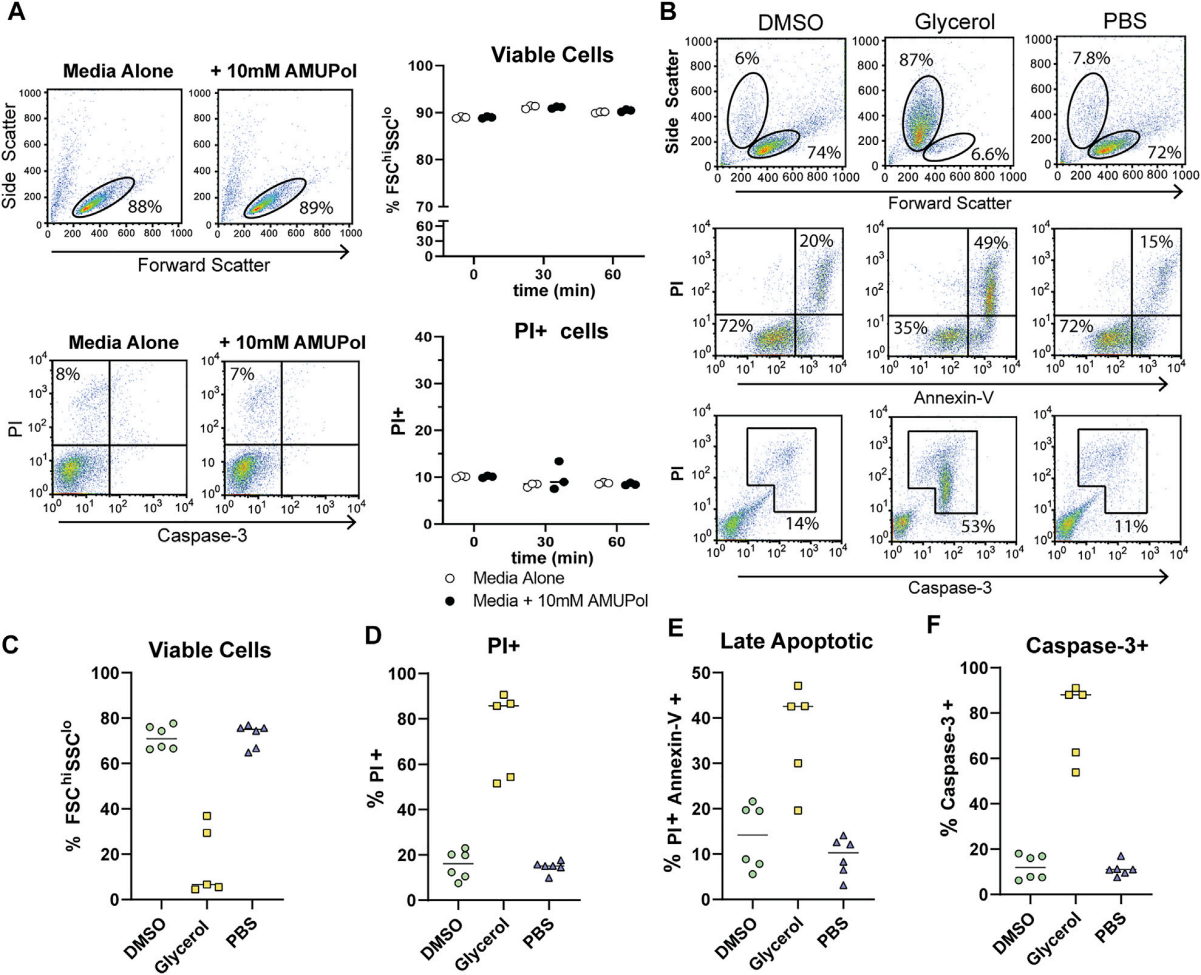
Cellular integrity and apoptosis in the presence of AMUPol and cryoprotecting agents. **(A)** Flow cytometric analysis of AMUPol treated cells. Flow cytometry plots are representative of three samples treated for 1 h. Percentages are determined based on the gates shown in the plots. Data is from a single experiment. **(B)** Representative flow cytometry plots of the effect of cryopreserving agents (in the absence of radical) on cell phenotypes and apoptosis. Percentages indicate the proportion of total cells within the indicated gates. Gates are set based on control (PBS treated) fluorescence intensities. All plots show total events. **(C)** Quantification of viable cells determined by FSC^hi^SSC^lo^ cells (top panels in B). **(D)** Quantification of PI positive cells as determined by gates shown in Supplementary Figure S1. **(E)** Quantification of late apoptotic cells determined by positive staining for PI and Annexin-V as shown in the upper right quadrant of the middle panels of **(B, F)** Quantification of caspase-3 positive cells determined by the gate shown in the bottom panels of (B). Data is pooled from 2 independent experiments.

In order to characterize the effect DNP preparation has on cell integrity and its relationship to DNP enhancements and spectral quality, we first assessed the cellular effects of DNP radicals and cryoprotecting reagents prior to cryogenic freezing. Comparison of the effects of AMUPol on cell size and viability by flow cytometry were carried out by incubating Jurkat T cells with 10 mM AMUPol for up to 1 h at 4°C. Cell viability was assessed using flow cytometry to measure forward light scatter (cell size), propidium iodide (PI) uptake (which reports on membrane permeability as the negatively charged PI can-not cross an intact plasma membrane) and caspase-3 cleavage (a marker of apoptosis). AMUPol had no effect on cell viability as determined by no change in the proportion of cells uptaking propidium iodide (PI) or exhibiting active caspase-3 cleavage (Figure 1A). Furthermore, there was no change in cell size [forward scatter intensity (FSC)] or cell density [side scatter intensity (SSC)], collectively indicating 10 mM AMUPol has no effect on membrane integrity or cellular homeostasis (Figure 1A).

The effects of cryopreserving media were then assessed after preparing cells for DNP in the absence of radical since the presence of 10 mM AMUPol had no effect on cell phenotypes. Cells were then incubated on ice for 10 min before staining for Annexin-V, caspase-3, and PI. Prior to the addition of cryoprotecting agent, cells were washed with PBS in D_2_O which has been demonstrated to increase radical T_2e_ and is correlated with increased DNP enhancements (Martorana et al., 2014; Sauvee et al., 2013). Exposing cells to D_2_O for short periods of time (within 24 h) has previously been shown to have no effect on cell morphology or viability (Martorana et al., 2014; Siegel et al., 1960). Incubation with 10% dimethyl sulfoxide (DMSO) had no observable effect on cell size or density with comparable FSC vs SSC profiles to control cells, which were treated with PBS (Figure 1B top panels and Figure 1C). PI uptake was not increased (Figure 1D) but there was a slight increase in late apoptotic cells compared to PBS treated cells, increasing from an average of 11–14% but as high as 20% in some samples (Figure 1B middle panels and Figure 1E) indicative of apoptosis activation. However, this was not accompanied by caspase-3 cleavage (Figure 1B bottom panels and Figure 1F), which shows the apoptotic phenotype induced by DMSO to be reversible (Kroemer et al., 2009).

The effects of glycerol preservation were tested by adding equal volumes of DNP matrix (60/30/10 glycerol/D_2_O/H_2_O) to the cell pellet, resulting in a final glycerol concentration of 30% v/v. Cells incubated with the glycerol mixture underwent significant dehydration in which cell size decreased by 50% and cell density increased by 50% (Figures 1B,C). Furthermore, glycerol induced significant late-stage apoptosis with 42% of cells staining for Annexin-V concomitant with a large increase in PI uptake and caspase-3 cleavage by cells exhibiting a dehydrated profile (Figures 1B–F), indicative of extensive dehydration and apoptosis prior to freezing. This establishes a significantly altered cellular phenotype and possibly structure of glycerol treated cells, prior to DNP analysis. On the other hand, DMSO induced significantly fewer perturbations prior to freezing.

### DNP Analysis of Cells Preserved With Different Cryoprotecting Formulations

**FIGURE 2:**
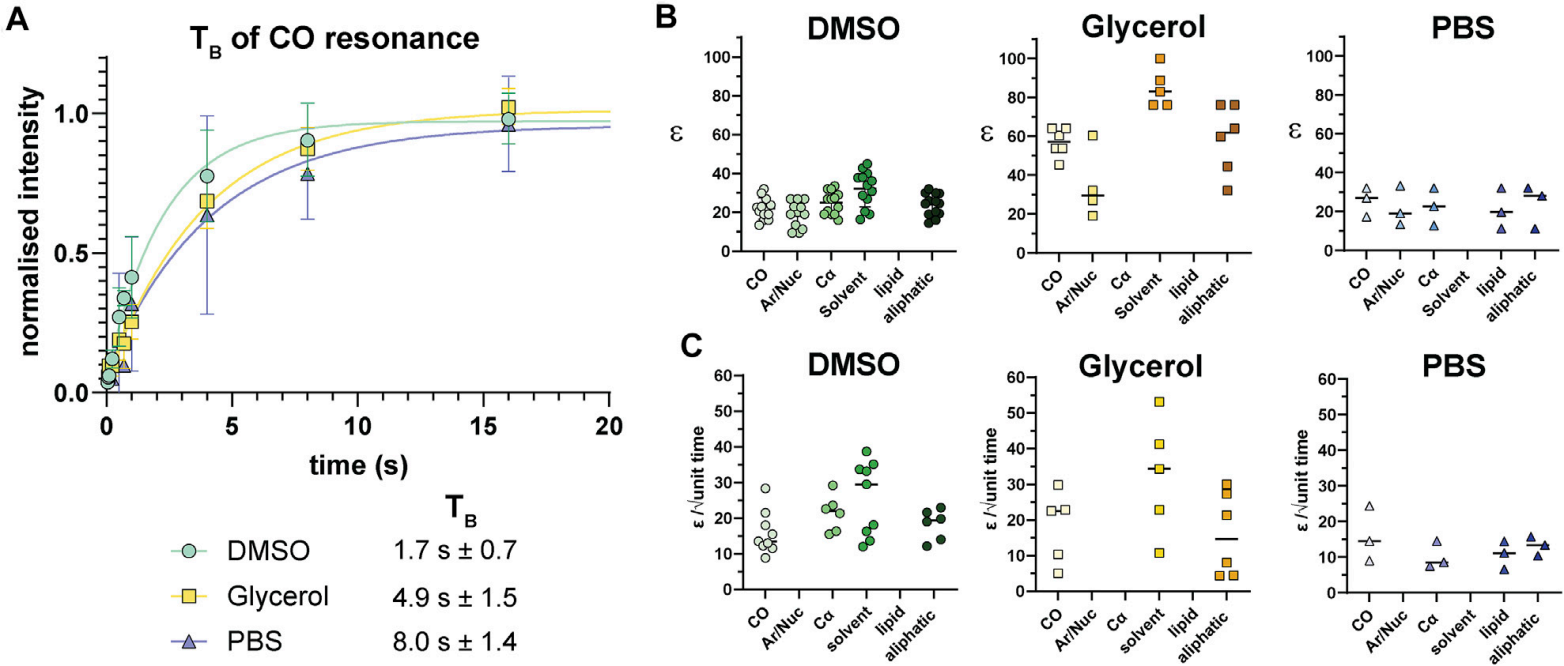
^13^C enhancements of Jurkat T cells. **(A)** T_B_ build up curves for carbonyl resonances with different cryoprotecting media. Error bars indicate the SD. **(B)** ^13^C enhancements (calculated as I_on_/I_off_ = ε_on/off_) in different cryoprotecting media across various cellular components as determined using data from the BMRB. **(C)** Enhancements calculated per √unit time using paired data from B and Supplementary Table I. Each symbol represents a single sample. Horizontal bars indicate the mean.

Following analysis of pre-freeze viability, we prepared unlabeled Jurkat T cells for DNP in cryopreservation media and a final radical concentration of 10 mM AMUPol. Samples were then flash-frozen in liquid nitrogen and the effects of different cryopreserving reagents on DNP enhancements were assessed. Throughout this work, a single sample refers to a single rotor containing ∼22 million cells. Comparison of polarization build up times (T_B_) of carbonyl resonances revealed cells preserved with glycerol exhibited a 2.9-fold longer T_B_ value of 4.9 s ± 1.5 compared to DMSO preserved cells at 1.7 s ± 0.7 and 8 s ± 1.4 for PBS preserved cells. (Figure 2A; Table 1). Initial comparison of cellular enhancements (determined by I_on_/I_off_ = ε_on/off_) showed glycerol preservation provided 2.6-fold greater ε_on/off_ of both solvent and cellular carbonyl (CO) signals compared to DMSO preserved cells (Figure 2B) and was mirrored in signal-to-noise ratios. However, taking into account T_B_ reveals glycerol preservation provides only a slightly larger enhancement per √unit time giving an average carbonyl enhancement of 18.1/√unit time ± 10 compared to 15.8/√unit time ± 6 for DMSO preserved cells and 16/unit time ± 7.8 for PBS preserved cells (Figure 2C). Interestingly, there was no correlation between T_B_ values and enhancements (Supplementary Figure S2) on a per sample basis, accounting for the spread in ε/√unit time. However, average values more closely resemble the linear relationship between ε_on/off_ and T_B_ predicted by simulations and observed experimentally by others (Mentink-Vigier et al., 2017). This is further emphasized by the sample variation in ε_on/off_ values observed for all cryopreservatives used. This could be reflective of heterogeneity in glass formation which may be a function of sample temperature history as glass homogeneity significantly contributes to DNP enhancements (Hall et al., 1997; Corzilius et al., 2014; Leavesley et al., 2018b). For frozen samples, significant unintentional temperature changes could be experienced during insertion/ejection from the probe and during capping (in the case of flash-frozen samples). Especially when using sapphire rotors due to the high thermal conductivity of sapphire. It is also likely that the ε_on/off_ values were additionally affected by variation in instrumentation performance such as microwave output and temperature regulation. We also measured quenching effects of AMUPol by acquiring in-cell spectra in the absence of radical. We found that in the presence of DMSO, 10 mM AMUPol results in a 50% reduction in the signal intensity of microwave off spectra, giving a quenching factor (ξ) of 0.5. In the presence of glycerol, the microwave off signal was quenched by 75% upon addition of AMUPol (Supplementary Figure S3), resulting in ξ of 0.25. If we take into account these measured quenching effects, then glycerol clearly performs poorly compared to 10% DMSO, giving an effective enhancement/√unit time of 4.5 s ± 2.5 and 7.9 s ± 3, respectively.

**TABLE 1:** T_B_ for different cellular resonances preserved under various preservation methods.

Resonance	Chemical shift	DMSO	Glycerol	PBS
Carbonyl (CO)	176 ppm	1.7 s ± 0.7	4.9 ± 1.5	8 s ± 1.4
Aromatic/Nucleic Acids	133 ppm	ND	ND	ND
Carbon α (Protein)	56 ppm	1.7 s ± 0.7	ND	6.6 s ± 0.6
Solvent	39.5 ppm (DMSO)	1.3 s ± 0.7	NA	NA
	65.2 ppm (Glycerol)	NA	3.2 s ± 1.0	NA
Lipid	34.5 ppm	ND	ND	6.6 s ± 0.6
Aliphatic	28 ppm	1.7 s ± 0.7	5 s ± 1.2	6.1 s ± 1.6
Number of samples		6	5	3

**FIGURE 3:**
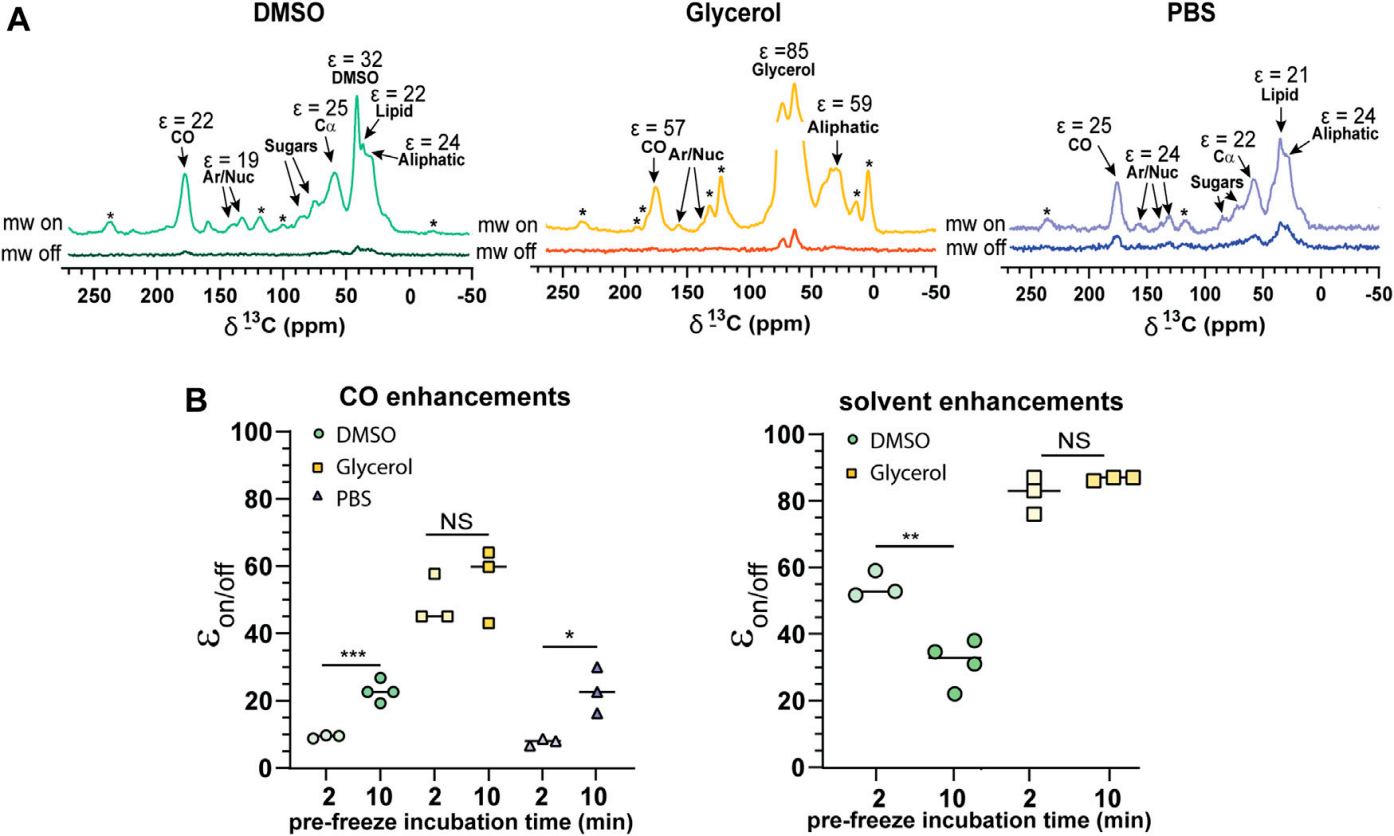
Cellular penetration of AMUPol in Jurkat T cells. **(A)** Comparison of ^1^H-^13^C CP spectra of 10% DMSO, glycerol, and PBS protected Jurkat T cells. Cellular components and their average enhancements are indicated. Enhancements are given as peak intensity ratios as not all signals could have the ε/√unit time determined. * indicates spinning side bands. **(B)** Enhancements obtained after 2 and 10 min incubations with AMUPol prior to flash-freezing for CO and solvent peaks. Statistical significance was determined using a parametric *t*-test where * = *p* < 0.05, ** = *p* < 0.01 and *** = *p* < 0.001, NS = not significant. DNP experiments were carried out at 600 MHz ^1^H larmor frequency at 9 kHz MAS.

We observed relatively uniform enhancements over various cellular components from CO, Cα, aromatics and lipids after incubating cells with 10 mM AMUPol for 10 min prior to freezing (Figure 3A; Supplementary Figure S4; Supplementary Table I) suggesting an even distribution of AMUPol with respect to different cellular components. Due to the dominance of the glycerol peak and its spinning side bands we were not able to identify differences in the enhancement of sugars, or Cα components in glycerol preserved cells but observed no difference in the enhancements of carbonyl and aliphatic components (Figure 3A). The exception was solvent ε_on/off_ values suggestive of increased radical concentration extracellularly. Consistent with this hypothesis, T_B_ values for solvent resonances was generally lower compared to cellular signals (Table 1). In addition, we also observed a time dependent component to the enhancements in DMSO treated cells but not glycerol treated cells. Incubating cells for 10 min prior to flash-freezing increased the enhancement of cellular signals by about 3-fold compared to those frozen after 2 min (Figure 3B). Concurrently, the solvent (DMSO) enhancement decreased from an average enhancement of ε_on/off_ = 52 after 2 min incubation to ε_on/off_ = 32 after 10 min, suggesting that diffusion of AMUPol into cells is delayed and solvent enhancements can be attributed to increased radical concentration outside the cell. This also implies that the concentration of AMUPol could be significantly increased to improve both ε_on/off_ and ε/√unit time values. The difference in time dependency of enhancements in DMSO prepared samples, compared to cells preserved with glycerol may be due to the effects of DMSO on membrane surfaces (Schrader et al., 2016). Accumulation of DMSO at the interfacial layer of the membrane may reduce membrane permeability to AMUPol. Certainly, the difference in dehydration effects of glycerol compared to DMSO would support this hypothesis. We did not observe an increase in enhancement after 10 min which may represent the time period required for the bulk cellular distribution of AMUPol (Supplementary Figure S5). This was also observed by Baldus and colleagues by microscopy using PyPol, a fluorescent variant of AMUPol (Narasimhan et al., 2019) where cellular fluorescence with PyPol peaked after 10–15 min.

### Post-DNP Cell Viability of Jurkat T Cells

**FIGURE 4:**
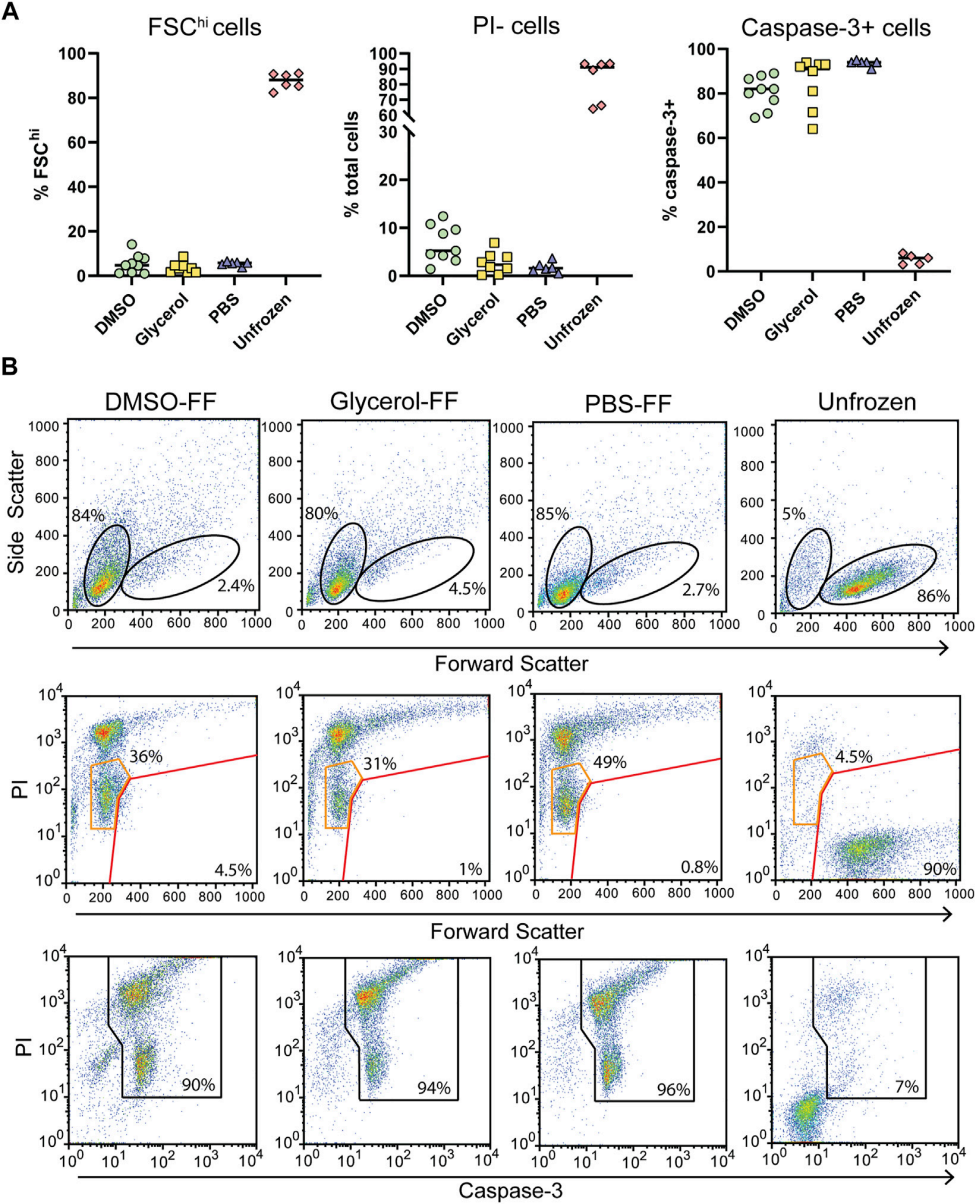
Post DNP cellular integrity and apoptosis induction with flash-freezing (FF). **(A)** Post-thaw viability as determined by FSC fluorescence, PI, and capsase-3 staining. Each symbol represents a single rotor. **(B)** Representative flow cytometry plots of samples treated with various cryoprotecting reagents followed by flash-freezing (FF). Top panels show the gating strategy used to determine FSC^hi^ cells. Middle panels show PI uptake of FSC^hi^ cells (red gate) and FSC^lo^ cells (orange gate) cells Bottom panels show the gating strategy used to determine caspase-3+ cells.

Following in-cell DNP, sample rotors were thawed by rapid warming in a 37°C water bath for 5 s and the cells immediately placed in culture for 24 h and assessed for viability. We observed low cell survival of flash-frozen cells, indicating poor vitrification across the ∼22 μL volume. Assessment of viability by trypan blue staining varied greatly between samples and also over-estimated the viability compared to the percentage of PI + cells detected by flow cytometry (Figure 4A; Supplementary Figure S6). Significant cell shrinkage and PI staining was observed with few to no viable PI- cells (Figure 4B). A significant population of PI^lo^FSC^lo^ cells were observed in frozen samples but not unfrozen samples (Figure 4B orange gates). These cells were predominantly caspase-3+, indicating that they are apoptotic and unlikely to be viable. Furthermore, viability was uncorrelated with cryoprotecting media as viability was equivalent to unprotected cells. Thus, the data clearly demonstrates poor cell viability of flash-frozen cells in 3.2 mm rotors. This would suggest significant intracellular ice crystal growth with this procedure.

**FIGURE 5:**
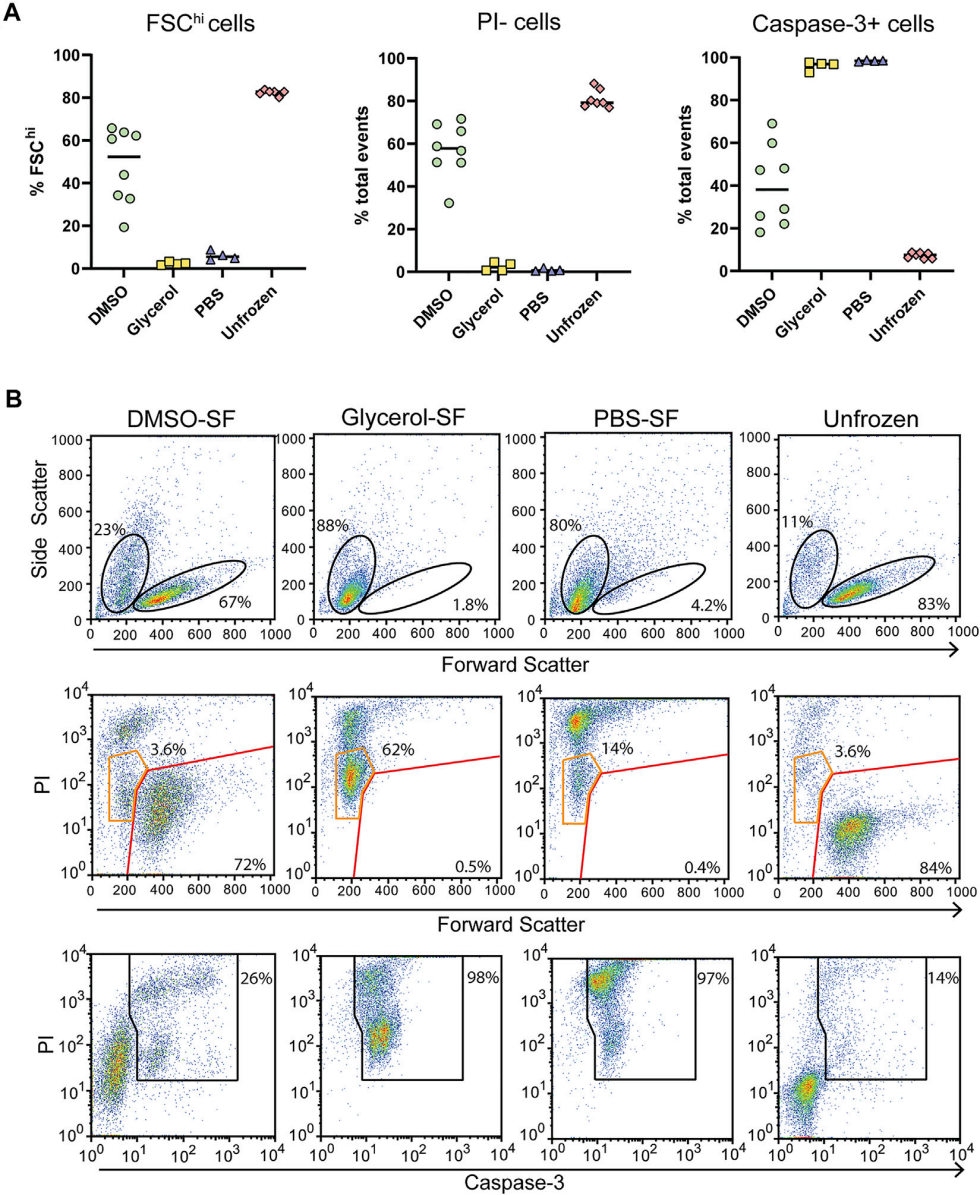
Post thaw cellular integrity and apoptosis induction with slow-freezing (SF). **(A)** Quantification of cell viability by flow cytometry through FSC fluorescence, PI, and caspase-3 staining. Each symbol represents a single sample/rotor. **(B)** Representative flow cytometry plots show the gating strategy used to determine the percentages in (A). All plots show total events. Top panels show the gating strategy used to determine FSC^hi^ cells. Middle panels show PI uptake of FSC^hi^ cells (red gate) and FSC^lo^ cells (orange gate) cells. Bottom panels show the gating strategy used to determine caspase-3+ cells.

For comparison, we assessed cells prepared the same way but that were control frozen with an approximate cooling rate of 1°C/min. Upon thawing only DMSO preserved cells benefited from slow-freezing with a significant increase in FSC^hi^ cells and reduced PI uptake (Figures 5A,B). Cell size and density was comparable to unfrozen cells and which generally correlates with increased viability. Glycerol and PBS preserved cells failed to recover from slow-freezing and exhibited equivalent FSC and SSC profiles to flash-frozen cells. In the case of glycerol preservation, PI fluorescence was lower, with most cells appearing as PI^lo^ compared to flash-frozen cells (Figure 5B orange gates). This was in contrast to PBS frozen cells which exhibited a predominantly PI^hi^ phenotype. Again, the viability of PI^lo^ cells is expected to be negligible at least 24 h post-thaw indicated by the low FSC fluorescence and high caspase-3 cleavage. However, the lower PI staining does indicate reduced membrane damage in the presence of glycerol, even if it is insufficient to preserve cell viability. Again, trypan blue staining over-estimated cell viability (Supplementary Figure S6). This could be due to cells faintly staining for trypan blue but remaining refractive under a light microscope. These cells likely take up PI and could form the PI^lo^ population we observed by flow cytometry. In addition, extreme dehydration caused by freezing results in a large reduction in cell size which could render cells too small to be recognized as cells by microscopy and are instead ignored as debris (both manually and with automated cell counters). This can be particularly challenging with a small cell type such as Jurkat T cells whose average size is 10 μm. These cells would shrink to <5 μm as indicated by flow cytometry, which is at or below the size limit for most automated cells counters and certainly likely to be difficult to distinguish by manual counting.

### DNP Analysis of Slow-Frozen Jurkat T Cells

**FIGURE 6:**
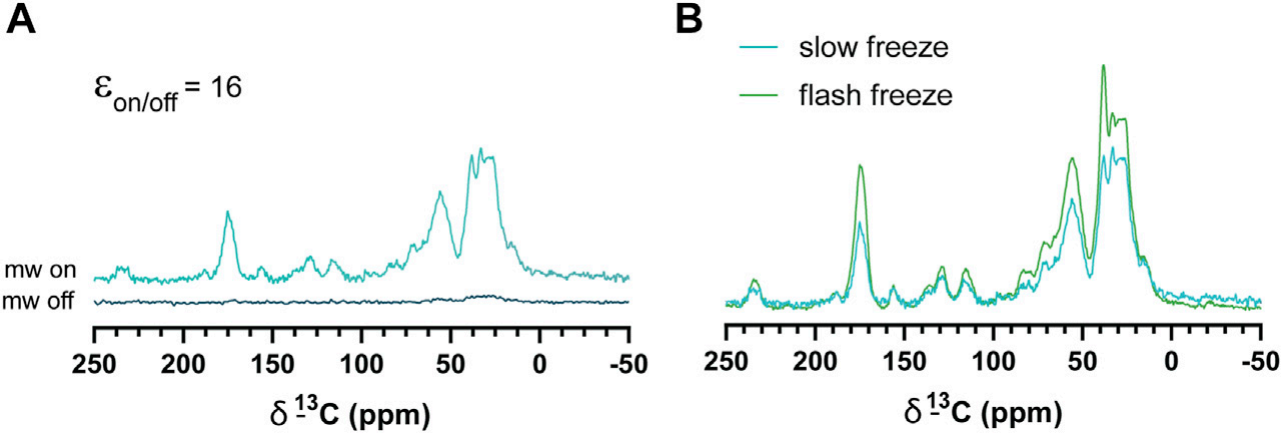
DNP enhancements of slow-frozen cells. **(A)** ^1^H-^13^C CP spectra of slow-frozen Jurkat T cells preserved with 10% DMSO. The enhancement given is the peak intensity ratio of the carbonyl resonance. **(B)** Comparison of microwave on spectra from slow-frozen cells in 10% DMSO (dark teal line) to flash-frozen cells in 10% DMSO (green line). DNP experiments were carried out at 600 MHz ^1^H larmor frequency at 9 kHz MAS.

Slow-frozen cells were also analyzed by DNP-NMR. We observed no significant spectral differences between slow-frozen and flash-frozen cells in 1D spectra. This was expected given the 1D spectra represent an average chemical shift of all cellular components and thus reports on chemical composition more so than structural features. (Figures 6A,B). The relative intensity of the DMSO peak was reduced compared to cellular signals in slow-frozen samples (Figure 6B). We think this reflects the accumulation of DMSO at the interfacial region of membranes reducing its accessibility to AMUPol, reducing net enhancements of DMSO (Liao et al., 2016; Schrader et al., 2016). Enhancements by peak intensity ratios were lower in slow-frozen cells compared to flash-frozen cells, although this was highly variable (Table 2). The calculated T_B_ time was increased in slow-frozen cells by an order of magnitude and consistent between the two samples assessed, averaging 17.3 s ± 1.4 for carbonyl resonances (Table 2). The calculated ε/√unit time was very low in slow-frozen cells, averaging 2.7/√unit time ± 0.3 compared to 12.7/√unit time ± 0.8 for flash-frozen cells. This data would be consistent with a lower radical concentration. It is unclear whether this is due to reduction of the radical during slow-freezing or a more even distribution of AMUPol resulting in reduced quenching effects but it is likely to be a combination of both.

**TABLE 2:** Average T_B_ values and enhancements (ε_on/off_) of slow-frozen Jurkat T cells compared to flash-frozen Jurkat T cells.

		Slow-frozen		Flash-frozen	
Resonance	Chemical shift (ppm)	TB	εon/off	TB	εon/off
Carbonyl (CO)	176	17.3 s ± 1.4	11 ± 6	1.75 s ± 0.2	17 ± 1.7
Aromatic/Nucleic Acids	133	17.1 s ± 1.1	ND	ND	ND
Carbon *a* (Cα)	56	17.7 s ± 0.4	12 ± 4	1.6 s ± 0.03	19 ± 3.3
Solvent (DMSO)	39.5	15.9 s ± 0.8	ND	1.1 s ± 0.1	27 ± 16
Lipid	34.5	15 s ± 0.6	ND	1.4 s ± 0.2	12 ± 1.2
Aliphatic	28	16.8 s ± 0.5	9 ± 2	1.5 s ± 0.2	20 ± 10
Number of Samples		2		3	

Certainly, the DNP performance of slow-frozen Jurkat T cells was markedly poorer compared to flash-frozen Jurkat T cells, despite their improved viability and raises the possibility that AMUPol may not be the optimal radical for in-cell DNP-NMR studies at least in Jurkat T cells or that significantly higher AMUPol concentrations are required, though this must be balanced with paramagnetic quenching effects. Alternatively, a move towards improved vitrification methods may prove more valuable.

## Discussion

In-cell DNP NMR is an impactful new Frontier in biomolecular NMR. The promise of physiologically relevant biomolecular structures and interactions is heavily complicated by the heterogeneity and compositional complexity of cellular samples. This poses an extreme challenge to atomic scale analyses such as NMR. The strong dependence of sample quality on the quality of subsequent NMR data highlights the need for rigorous studies of cell preparation methods for in-cell DNP-NMR and especially given the added complication of cryogenic temperatures. The work presented here provides a significant contribution to this analysis and highlights key considerations when performing in-cell DNP-NMR towards improved *in situ* structural studies.

The goal of in-cell NMR is to capture native biomolecular conformations at the time of analysis. This is under the reasonable assumption that conformational relevance can be linked with cell viability. Cell viability after cryogenic freezing is affected in two independent ways. The first is damage done during freezing or by cryoprotectants (prior to analysis) and the second is damage done during warming (after analysis). Of most relevance to in-cell DNP is damage done prior to or during freezing as this establishes the conformational landscape of the sample at the time of analysis. Our study of pre-freeze viability demonstrates 30% glycerol (mimicking the use of DNP matrix for sample preparation) to be highly detrimental to cell viability and membrane integrity. Cells became extremely dehydrated and apoptotic within 10 min of glycerol exposure and thus significant damage occurs prior to freezing. Under the conditions tested here, glycerol preservation significantly alters the molecular state of cells analyzed by DNP. We detected apoptosis by Annexin-V staining in which fluorophore labelled Annexin-V binds to phosphotidylserine on the outer leaflet of the plasma membrane (Lakshmanan and Batra 2013). Phosphotidylserine does not localize to the outer leaflet of healthy mammalian cells but begins to accumulate in the outer leaflet of apoptotic cells. The induction of apoptosis and alterations to the lipid composition of the plasma membrane should be an important consideration when choosing a DNP preparation method. This is a crucial consideration for the study of proteins with charge-charge interactions or lipid interactions, as this may create a population of proteins whose interactions are biologically irrelevant or only relevant to apoptotic processes.

Following freezing, we found survivability to be poor in flash-frozen cells. Viability was largely uncorrelated with the presence of cryopreserving reagents under flash-freezing methods. This clearly indicates a lack of vitrification of flash-frozen 3.2 mm rotors. Cooling rates of 10°C/s have been measured for liquid nitrogen flash-frozen 3.2 mm rotors (Hu et al., 2009). Estimated cooling rates required to obtain perfect vitrification are in excess of 10,000°C/s depending on the concentration of cryoprotectant and as high as 10^6^°C/s for pure water (Berejnov et al., 2006), which is difficult to achieve with a 3.2 mm rotor of ∼22 μl. High concentrations of protein or cryoprotectant can increase the glass transition temperature, which reduces the cooling rate required to achieve vitrification. Intracellular ice crystal growth is thought to be the greatest contributor to cell death by cryogenic damage and which occurs above the glass transition temperature (Mazur 2004). Studies using differential scanning calorimetry (DSC) suggest that liquid nitrogen freezing of samples above the intracellular glass transition results in poor cell survival, as was also observed in our study (Meneghel et al., 2019). The authors provide evidence for an intracellular transition of around −50°C (Meneghel et al., 2019). Thus, vitrification of the intracellular compartment requires fast cooling rates down to −50°C. On the other hand, significantly improved post-thaw cell viability was achieved with slow-freezing at the typical 1°C/min but only for DMSO preserved cells. Glycerol preserved cells did not survive slow or flash-freezing with recovery of viable cells comparable to cells frozen in the absence of cryoprotectant. Presumably, the poor viability of glycerol preserved cells in this case is due to the dehydrating and apoptotic effects prior to cells entering the frozen state. Thus, for increased viability, in-cell DNP samples require slow-cooling methods and DMSO as cryoprotectant.

But is cell viability correlated with structural relevance? DMSO is known to have effects on membrane phase behavior and hydration, having been demonstrated to out complete water for hydrogen bonding of lipid head-groups, at least in model membrane systems (Schrader et al., 2015). Furthermore, room temperature studies of model membrane systems suggest that DMSO improves protein conformational homogeneity for structural studies, however this also implies non-physiological conformational selection (Liao et al., 2016) and could be due to the effects of DMSO on lipid chain melting temperature and interfacial hydration. Thus, while DMSO greatly improves cell viability, it may not necessarily lead to physiological structures. On the other hand, glycerol has not been documented to affect the biophysical properties of model membrane systems. Despite this, we observed compositional changes of the plasma membrane in the presence of glycerol in addition to severe dehydration effects. Dehydration is problematic for protein structural studies as the loss of water can greatly affect protein conformation due to the critical role played by solvent water in driving protein folding (Dill 1990; Wolkers et al., 2019). The dysregulation of electrolyte and small molecule concentrations should also be considered when performing in-cell structural studies and the effects of dehydration on ligand interactions and charge-charge interactions. These effects would be particularly problematic for drug interaction studies. Cellular dehydration is also significant in cells frozen slowly (Meneghel et al., 2019) as described earlier. This gives rise to freeze concentration of intracellular solutes and has been shown to enhance ROS production (Len et al., 2019), and activation of apoptotic pathways during the freezing process until the intracellular glass transition temperature is reached (Bissoyi and Pramanik 2014). These effects could be minimized with vitrification.

Vitrification may be achievable in smaller rotors such as 1.3 mm (2.5 μl volume) or 0.7 mm (0.56 μl volume) outer diameter rotors, particularly as higher spinning frequencies can now be reached under DNP conditions (Chaudhari et al., 2016; Berruyer et al., 2020). Given the widespread use of pulled straws of 1.7–0.8 mm in the vitrification of human embryonic stem cells and blastocytes in liquid nitrogen (with excellent post-thaw viability) this appears eminently possible (Chen et al., 2001). Thus, for high resolution structural studies, slow-cooling or cryoprotectants may not be suitable for the preservation of native membrane structures or transiently assembled signaling complexes for DNP analysis, despite improved viability post-DNP. It may be that like *in situ* cryo-EM, where the use of vitrification methods that obtain higher resolution images are favored over higher viability methods, so to structural studies by in-cell DNP-NMR might also have to balance the need for resolution and structural relevance with viability. Therefore, given the effects of cryopreserving reagents and slow-freezing on molecular structure and composition documented in this study and by others, it seems prudent to develop methods towards sample vitrification and cryoprotecting reagent free in-cell DNP-NMR.

We also observed distinctions between cryoprotectants in DNP parameters. Enhancements per √unit time were only slightly better in glycerol protected cells compared to DMSO preservation. This is attributable to the longer polarization build up time of glycerol preserved cells, despite exhibiting a 2.6-fold larger ε_on/off_ compared to DMSO prepared cells. Shorter T_B_ times with d_6_-DMSO is an interesting observation and it is unclear whether additional cell specific factors are contributing to such a short T_B_ time or whether the distribution of DMSO compared to glycerol influences this value or its effect on ice formation. The use of d_6_-DMSO precludes the contribution of methyl mediated cross-relaxation to the short T_B_ time. The effect of fast polarization build up with lower enhancement factors in the presence of DMSO has also been reported in model membrane systems (Liao et al., 2016) and so it is unclear whether this is a property of AMUPol-DMSO interactions or a specific property of d_6_-DMSO glasses. The variation in enhancement factors of glycerol mixtures <55% v/v in model systems are thought to be due to increased water-water interactions, presumably manifesting as increased ice formation (Leavesley et al., 2018b). Ice crystalization is also thought to increase paramagnetic quenching of nuclei by freeze concentrating DNP radicals outside of growing ice crystals which are extracting water from the surrounding space to feed crystal growth (Corzilius et al., 2014). The resulting increase in e-e-n polarization transfer at higher radical concentrations leads to reduced enhancement factors and shorter polarization build up times (Leavesley et al., 2018a). While the lower T_B_ time of DMSO preserved samples would be consistent with increased radical concentration, possibly due to ice formation, we would expect this phenomenon to be greater in PBS preserved cells in which ice crystal formation would be expected to be greatest. Since polarization build up times in PBS treated samples were consistently longer than that observed in DMSO preserved cells it suggests ice crystalization is not driving this phenomenon. The greatly increased T_B_ time of slow-frozen cells suggest that longer T_B_ times are reflective of lower cellular radical concentrations. While differences in ice formation can-not be ruled out, it seems much more likely that slow-frozen cells have lower AMUPol concentrations due to reduction of AMUPol by the intracellular environment and/or improved distribution of AMUPol throughout the cells. In line with this hypothesis, net enhancements were generally lower in slow-frozen cells, suggestive of reduced radical concentration, although the measured values were too variable to make a convincing conclusion and requires further investigation. Consistent with this hypothesis, solvent signals consistently exhibited shorter T_B_ times and higher enhancements in flash-frozen samples, which could be explained by a higher concentration of AMUPol and DMSO outside the cell compared to inside the cell. Although this does not necessarily provide a clear demarcation between intracellular and extracellular signals. One might expect that T_B_ times of cellular signals to exhibit a wider range in values if this was the case. However, the ambiguity of intracellular verses extracellular signals makes such an assertion difficult. The T_B_ of extracellular glyco-proteins and plasma membrane signals could account for much of the cellular signal observed at earlier time points and longer relaxing intracellular signals may be hidden within the bulk build up curves.

## Conclusion

The use of glycerol and its perturbation of cellular integrity and apoptosis induction makes glycerol not optimal for in-cell DNP. In addition, the enhancements per √unit time are only marginally greater than those achieved through using d_6_-DMSO. Overall, we greatly favor the use of DMSO. A slow-freeze (1°C/min) protocol for the preparation of Jurkat T cells for DNP analysis is preferable when viability is a priority. However, the optimal cooling rate is likely dependent on cell type and intracellular localization of molecules of interest. Furthermore, the impact of cooling rates and cryoprotectants on protein/membrane structures remains to be determined. It is likely that the benefits of slow-freeze protocols will be diminished with smaller rotors if sufficiently fast cooling rates can be achieved with flash-freezing to reduce cellular dehydration through improved vitrification, potentially in the absence of cryoprotecting agents. These challenges highlight the need for ongoing studies in this area and the investigation of vitrification methods for in-cell DNP.

## Data Availability

The raw data supporting the conclusion of this article will be made available by the authors, without undue reservation.
