# Supplementary material for: Biomolecular Perturbations in In-Cell Dynamic Nuclear Polarization Experiments
Source: Front Mol Biosci. 2021 Oct 21;8:743829. doi: 10.3389/fmolb.2021.743829 (PMC8572051; doi:10.3389/fmolb.2021.743829)
Supplement: Supplementary file 1 [file DataSheet1.PDF]

Supplementary Information

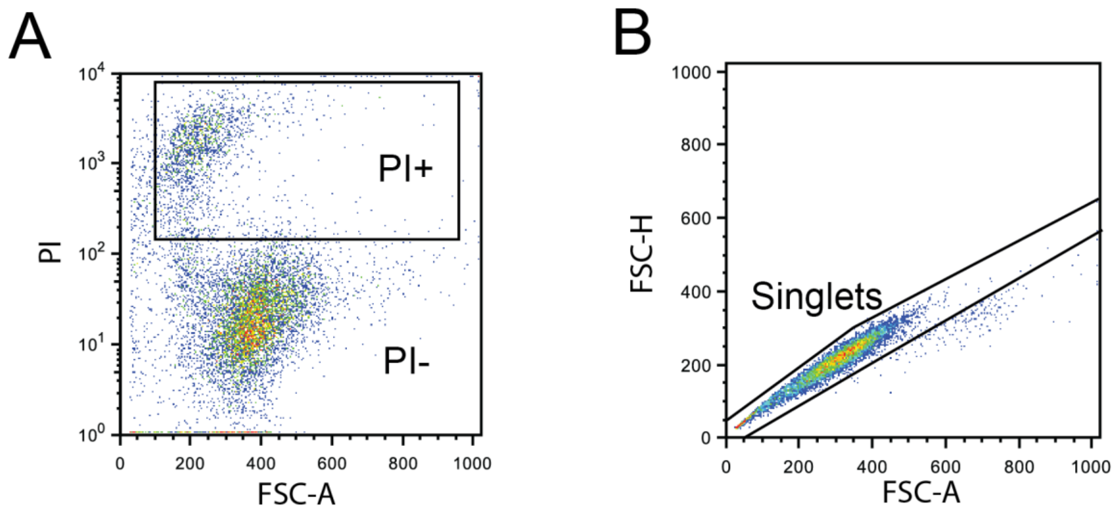

Supplementary Figure S1: **Flow cytometry gating strategy.** A) Determination of PI+ cells. B) Gating strategy for removing doublets from each plot. The cells within the gate shown are singlets and are used for further analysis. All plots shown throughout this work are gated to removed doublets as show above.

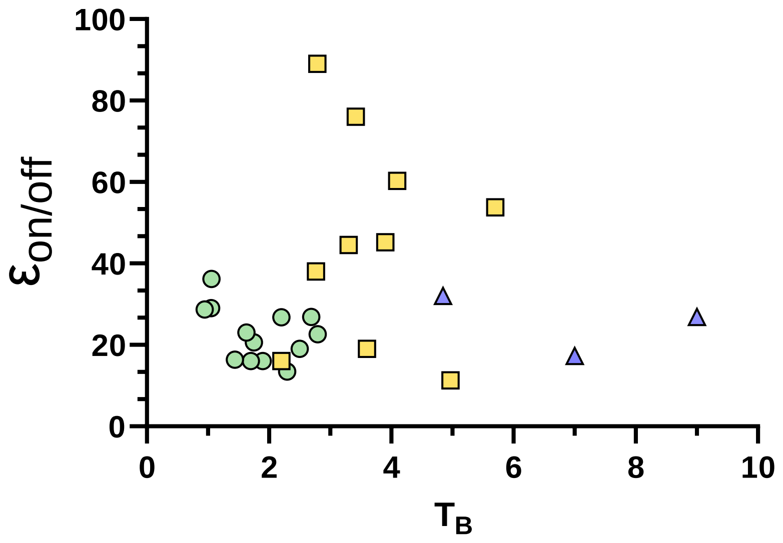

Supplementary Figure S2: Correlation between enhancements ($\varepsilon_{on/off}$) with polarization build up times (T_B). Each dot represents either a solvent or CO resonance. Green dots indicate DMSO preserved cells (6 samples) and yellow squares indicated glycerol preserved cells (5 samples) and blue triangles are PBS preserved cells (3 samples).

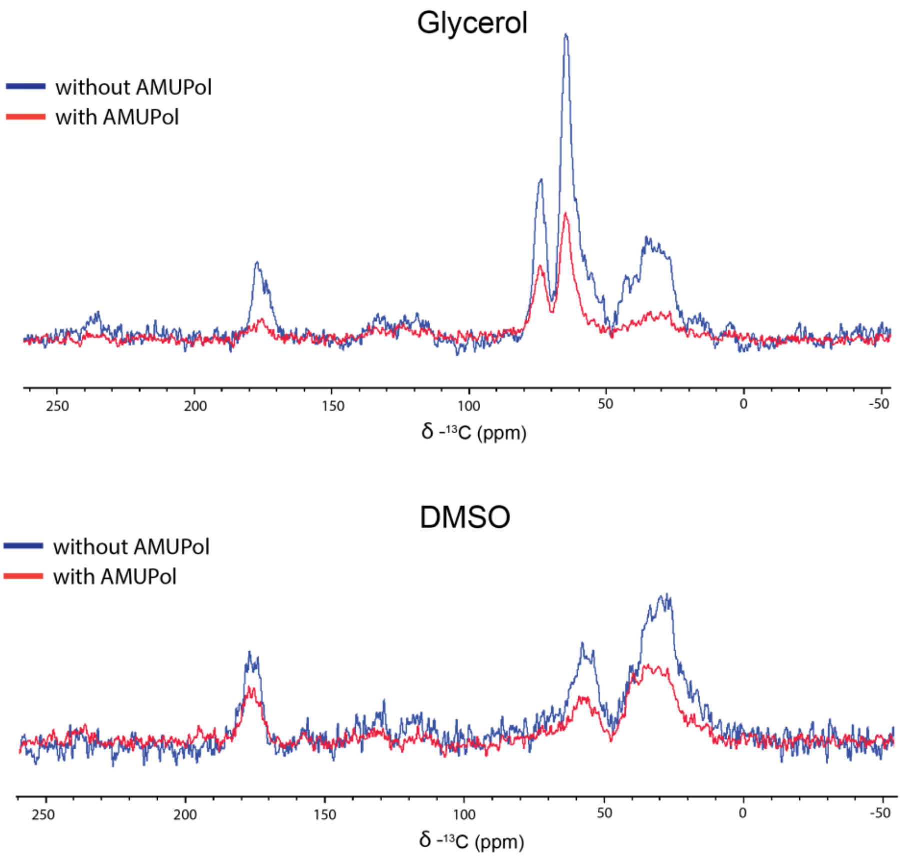

Supplementary Figure S3: Signal quenching of AMUPol in in-cell DNP-NMR.
^{1}H-^{13}C CP spectra of Jurkat T cells at 100 K with 10 mM AMUPol (red lines) or without AMUPol (blue lines). Data shown is from a single sample of DMSO preserved cells and a single sample of glycerol preserved cells. Both spectra were acquired with 512 scans and a recycle delay of 1.26s*T1. In the case of samples without radical, this equates to 82 s delay for both DMSO and glycerol preserved samples, while in the presence of radical the recycle delay was 2 s for DMSO preserved samples and 6 s for glycerol preserved samples.

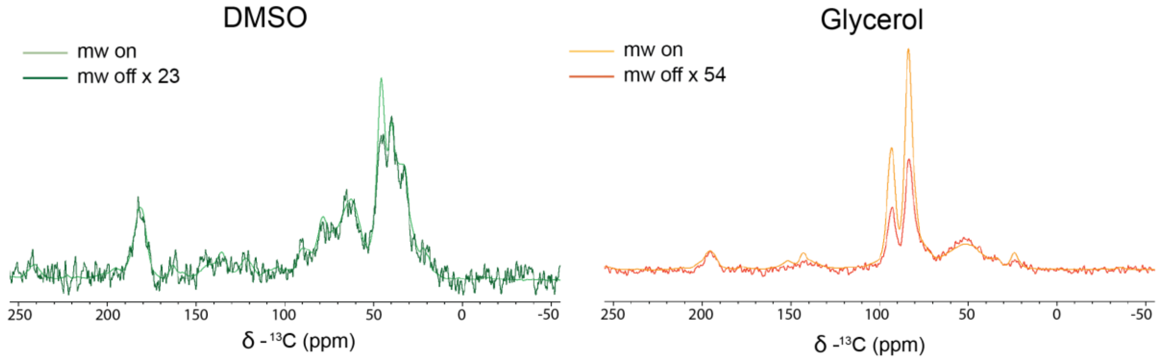

Supplementary Figure S4: ^{1}H-^{13}C CP MAS spectra of Jurkat T cells. Microwave on and off spectra scaled to the carbonyl resonance are shown. Data is representative of 13 samples (DMSO treated) and 6 samples (glycerol treated). Data is shown to indicate the signal-to-noise of the data analysed and highlight regions of the spectrum that were difficult to determine enhancement values due to limited signal-to-noise (particularly in the aromatic region).

Supplementary Table I: Raw signal intensity data used in calculating DNP enhancements shown in Figure 2.

Sample	CO I_{on}	CO I_{off}	ε	Ar I_{on}	Ar I_{off}	ε	Ca I_{on}	Ca I_{off}	ε
DMSO	169992.23	7210.45	23.6	52500.26	2674.22	19.6	208082.62	7894.63	26.4
DMSO	134118.34	7918.16	16.9	39057.88	2705.88	14.4	165207.65	6059.45	27.3
DMSO	68708.62	3641.31	18.9	11931.6	602.95	19.8	78848.67	2900.26	27.2
DMSO	64468.74	3103.46	20.8	6888.26	730.11	9.4	78239.59	2729.7	28.7
DMSO	73098.29	3050.07	24.0	5727.22	571.1	10.0	82122.92	3924.02	20.9
DMSO	87341.08	2987.41	29.2	23820.36	1151.97	20.7	91941.22	2819.55	32.6
DMSO	381631.32	16502.07	23.1	100519.23	4291.29	23.4	641526.3	29571.29	21.7
DMSO	74166.84	2233.6	33.2	26957.73	1031.96	26.1	77702.54	2162.23	35.9
DMSO	77662.02	3368.94	23.1	22902.09	851.49	26.9	86217.82	2704.2	31.9
DMSO	51145.87	2394.37	21.4	11889.28	1515.66	7.8	55085.62	2921.63	18.9
DMSO	41354.5	2723.12	15.2	55565.37	2110.98	26.3	48855.75	3169.36	15.4
DMSO	213767.41	11787.88	18.1	63534.64	2967.04	21.4	219671.45	11076.81	19.8
DMSO	103419.12	9709.92	10.7				184533.75	9943.12	18.6
Glycerol	282361.41	4439.28	63.6	84694.86	3132.16	27.0			
Glycerol	256958.36	4003.35	64.2	61234.86	3043.44	20.1			
Glycerol	351778.8	6724.41	52.3	76341.43	2355.14	32.4			
Glycerol	344283.83	7669.58	44.9	112108.57	1884.95	59.5			
Glycerol	340250.81	6267.88	54.3						
Glycerol	445529.13	7322.96	60.8						
PBS	64115.91	3691.33	17.4	27372.37	1367.51	20.0	25569.14	2090.29	12.2
PBS	316851.66	9968.02	31.8	76535.9	2298.7	33.3	945246.41	29491.77	32.1
PBS	39747.61	1421.11	28.0	9423.7	735.42	12.8	41100.3	1827.17	22.5

Sample	Solvent I_{on}	Solvent I_{off}	ε	lipid I_{on}	Lipid I_{off}	ε	Aliphatic I_{on}	Aliphatic I_{off}	ε
DMSO	1113394.61	29974.69	37.1				265289.54	10926.22	24.3
DMSO	1033111.58	34461.99	30.0				229518.58	12689.1	18.1
DMSO	474693.02	13343.42	35.6				101772.51	4743.91	21.5
DMSO	459484.71	20552.5	22.4				93399.55	4942.27	18.9
DMSO	221006.07	5725.35	38.6				142531.88	4512.39	31.6
DMSO	270197.94	6286.21	43.0				144461.03	4902.34	29.5
DMSO	525116.53	20425.95	25.7				710281.84	29327.43	24.2
DMSO	233123.19	5695.26	40.9				125441.72	4939.53	25.4
DMSO	237743.02	6488.38	36.6				131536.65	4874.17	27.0
DMSO	399945.87	13103.48	30.5				86173.74	5136.53	16.8
DMSO	59026.68	3544.38	16.7				69238.44	5262.76	13.2
DMSO	870764.78	19446.21	44.8				310630.55	10661.48	29.1
DMSO	389519.15	21212.92	18.4				605053.02	18946.47	31.9
Glycerol	8496610.14	86572.49	98.1				280850.12	3698.46	75.9
Glycerol	7991043.45	98168.08	81.4				287159.97	4492.9	63.9
Glycerol	3411107.34	44385.69	76.9				517906.3	11509.43	45.0
Glycerol	4080349.51	46102.45	88.5				473033.27	15098.28	31.3
Glycerol	2145319.48	27965.66	76.7				488226.82	6350.18	76.9
Glycerol							790616.41	12976.16	60.9
PBS				57149.05	4841.27	11.8	42119.59	3551.94	11.9
PBS				386452.59	13971.24	27.7	516169.19	16063.76	32.1
PBS				88786.28	4602.22	19.3	59774.17	2110.73	28.3

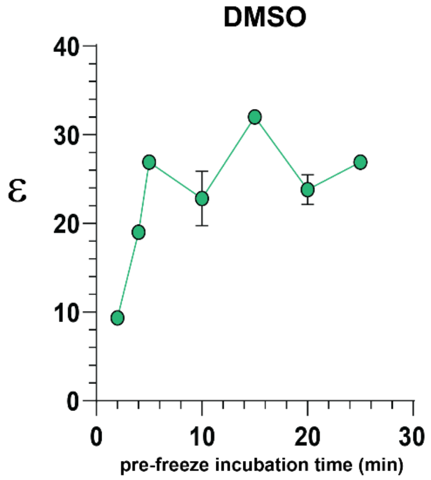

Supplementary Figure S5: Cellular enhancements as a function of incubation time. Jurkat T cells cryopreserved with 10% DMSO and 10 mM AMUPol. After incubation at the times indicated, cells were frozen into DNP rotors. Error bars indicate the SD. At 2 min, n = 3, 4 min n = 1, 5 min n= 1, 10 min n = 4, 15 min n = 1, 20 min n = 3 and 25 min n = 1.

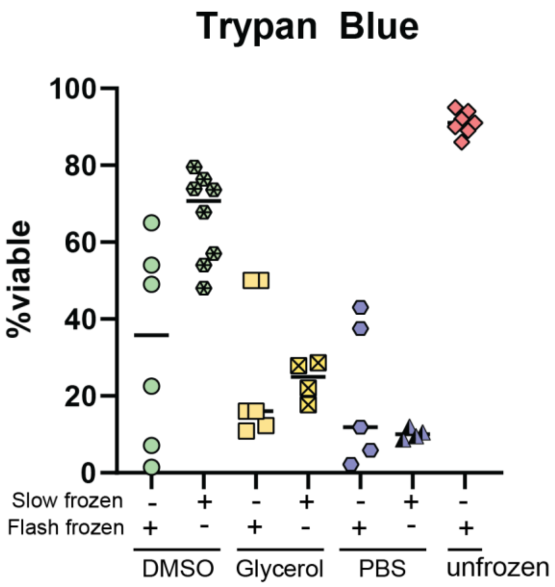

Supplementary Figure S6: Viability determined by trypan blue staining in slow frozen and flash frozen cells. The data here corresponds to the rotors analysed by flow cytometry presented in Figure 4 and 5. Viability was determined 24 h after thawing at the same time cells were analysed by flow cytometry.
